# The Effectiveness of Exercise Physiology Services During the COVID-19 Pandemic: A Pragmatic Cohort Study

**DOI:** 10.1186/s40798-022-00539-3

**Published:** 2023-01-08

**Authors:** Patrick J. Owen, Shelley E. Keating, Christopher D. Askew, Kelly M. Clanchy, Paul Jansons, Ralph Maddison, Andrew Maiorana, Jenna McVicar, Suzanne Robinson, Christopher Neason, Matthew J. Clarkson, Niamh L. Mundell

**Affiliations:** 1 Institute for Physical Activity and Nutrition, School of Exercise and Nutrition Sciences, Deakin University, Geelong, VIC Australia; 2 School of Human Movement and Nutrition Sciences, The University of Queensland, St Lucia, QLD Australia; 3 VasoActive Research Group, School of Health and Behavioural Sciences, University of the Sunshine Coast, Sippy Downs, QLD Australia; 4 Sunshine Coast Health Institute, Sunshine Coast Hospital and Health Service, Birtinya, QLD Australia; 5 School of Health Sciences and Social Work, Griffith University, Gold Coast, QLD Australia; 6 Menzies Health Institute, Griffith University, Gold Coast, QLD Australia; 7 Department of Medicine, School of Clinical Sciences at Monash Health, Monash University, Clayton, VIC Australia; 8 Allied Health Department, Fiona Stanley Hospital, Perth, WA Australia; 9 Curtin School of Allied Health, Curtin University, Perth, WA Australia; 10 Curtin School of Population Health, Curtin University, Perth, WA Australia; 11 Deakin Health Economics, Institute for Health Transformation, Deakin University, Geelong, Australia; 12 Better Exercise Physiology, Healesville, VIC Australia; 13 Institute for Health and Sport, Victoria University, Melbourne, VIC Australia

**Keywords:** Telehealth, Coronavirus, Telemedicine, eHealth, Rehabilitation

## Abstract

**Background:**

The COVID-19 pandemic markedly changed how healthcare services are delivered and telehealth delivery has increased worldwide. Whether changes in healthcare delivery borne from the COVID-19 pandemic impact effectiveness is unknown. Therefore, we examined the effectiveness of exercise physiology services provided during the COVID-19 pandemic.

**Methods:**

This prospective cohort study included 138 clients who received exercise physiology services during the initial COVID-19 pandemic. Outcome measures of interest were EQ-5D-5L, EQ-VAS, patient-specific functional scale, numeric pain rating scale and goal attainment scaling.

**Results:**

Most (59%, *n* = 82) clients received in-person delivery only, whereas 8% (*n* = 11) received telehealth delivery only and 33% (*n* = 45) received a combination of delivery modes. Mean (SD) treatment duration was 11 (7) weeks and included 12 (6) sessions lasting 48 (9) minutes. The majority (73%, *n* = 101) of clients completed > 80% of exercise sessions. Exercise physiology improved mobility by 14% (*β* = 0.23, *P* = 0.003), capacity to complete usual activities by 18% (*β* = 0.29, *P* < 0.001), capacity to complete important activities that the client was unable to do or having difficulty performing by 54% (*β* = 2.46, *P* < 0.001), current pain intensity by 16% (*β* = − 0.55, *P* = 0.038) and goal attainment scaling t-scores by 50% (*β* = 18.37, *P* < 0.001). Effectiveness did not differ between delivery modes (all: *P* > 0.087).

**Conclusions:**

Exercise physiology services provided during the COVID-19 pandemic improved a range of client-reported outcomes regardless of delivery mode. Further exploration of cost-effectiveness is warranted.

**Supplementary Information:**

The online version contains supplementary material available at 10.1186/s40798-022-00539-3.

## Key Points

- During the COVID-19 pandemic, most clients received exercise physiology services via in-person delivery only, rather than telehealth only.
- Exercise physiology services provided during the COVID-19 pandemic increased functional capacity, reduced pain intensity and resulted in clients achieving their treatment-based goals.
- Changes in client-reported outcome measures did not differ by delivery mode.

## Introduction

The Coronavirus Disease 2019 (COVID-19) pandemic has led to unprecedented challenges for healthcare systems. To control the transmission of COVID-19, physical distancing measures were commonplace [1]; however, these measures complicated the provision of healthcare traditionally reliant on in-person delivery [2]. A burgeoning strategy for the provision of healthcare was telehealth delivery, which is conceptually defined as remote services between the practitioner and client either asynchronously (delayed interaction; e.g. email) or synchronously (real-time interaction; e.g. video calls) [2]. Telehealth delivery of healthcare services increased in response to the COVID-19 pandemic worldwide [3]. For example, a recent audit of exercise physiology services in Australia demonstrated that following the onset of the COVID-19 pandemic, 91% of clinicians offered some form of telehealth delivery, compared to 25% prior [4]. Therefore, it is pertinent to determine whether changes in delivery mode borne from the COVID-19 pandemic have impacted healthcare treatment effectiveness.

Exercise physiology is an established therapeutic service for myriad chronic conditions (e.g. psychiatric, neurological, metabolic, cardiovascular, pulmonary, musculoskeletal, cancer) and has traditionally relied upon in-person interactions between exercise physiologists (tertiary qualified allied health professionals who specialise in the prescription of exercise for a range of chronic and complex conditions and in Australia are accredited by Exercise and Sports Science Australia as the professional regulatory body) [5] and clients [6]. Evidence also supports exercise physiology may be superior to other conservative therapies of chronic low back pain [7] and may also be a viable conservative alternative to surgery for femoroacetabular impingement syndrome [8]. However, the vast majority of evidence supporting the efficacy of exercise physiology is from studies conducted in research settings (rather than clinical or industry settings) that investigated in-person delivery modalities only. Given the exercise physiology profession rapidly adopted both synchronous and asynchronous telehealth delivery (also known as ‘tele-exercise’ [9]) as a sole or adjunct to in-person delivery to continue provision of services during the COVID-19 pandemic [10], evaluating whether these changes in service impacted effectiveness is warranted.

The primary aim of this study was to determine the effectiveness of exercise physiology services provided during the COVID-19 pandemic (July 2020 to June 2021) on client-reported outcome measures. It was hypothesised that exercise physiology services provided during the COVID-19 pandemic would improve client-reported outcome measures regardless of method of delivery. Secondary aims compared the effectiveness and financial costs between exercise physiology services provided via in-person delivery only, telehealth delivery only and a combination of both in-person and telehealth delivery.

## Methods

### Study Design and Setting

We conducted a prospective pragmatic cohort study examining the effectiveness and financial costs of exercise physiology services during the inaugural year of the COVID-19 pandemic (after 25 January 2020; date of first confirmed case in Australia). The data collection period spanned 22 July 2020 to 30 June 2021 and included 138 client datasets. The study was conducted in line with the National Statement on Ethical Conduct in Human Research (2007) and the Declaration of Helsinki. The study was approved by Deakin University Human Ethics Advisory Group – Health (90-2020-200512). All client and clinician participants provided informed written consent prior to involvement in the study.

### Participants

Participants were clients of accredited exercise physiology clinicians receiving exercise physiology services during the COVID-19 pandemic. Potential participants were primarily sought by targeting clinicians through social media advertisement (e.g. Twitter and Facebook), newsletters and magazines periodically released by Exercise and Sports Science Australia, as well as via word-of-mouth through the professional networks of the study authors. Clinicians were invited to share details of the study with their clients, who then independently decided whether to participate in the study. Clinicians and clients from all states/territories of Australia were eligible to participate. No restrictions on clinician or client demographics were employed.

### Data Collection

Clinicians provided anonymised client datasets via an online survey (Qualtrics, Provo, UT) at the beginning and end of an exercise physiology treatment phase. Given the pragmatic nature of this study, there was no minimum or maximum treatment phase duration and this was at the discretion of the clinician for each individual client. Data collected were: client demographics, exercise physiology treatment details, client-reported outcome measures and adverse events.

#### Health-Related Quality of Life

The EQ-5D-5L is a five-item questionnaire that measures health across five dimensions: (1) mobility, (2) self-care, (3) usual activities, (4) pain/discomfort, and (5) anxiety/depression [11]. Each dimension contains five levels that identify either no problems, slight problems, moderate problems, severe problems or unable to/extreme problems. The EQ-VAS is a single-item visual analogue scale spanning 0–100 points that measures overall health. Zero represents the worst health imaginable and 100 signifies the best health imaginable. Health effects measured using the EQ-5D-5L were converted to an overall utility score using an established UK value set [12]. The EQ-5D-5L has moderate-good test–retest reliability (ICC = 0.73–0.84), whereas the EQ-VAS has moderate test–retest reliability (ICC = 0.61–0.68) [13].

#### Functional Capacity

The patient-specific functional scale is a single-item scale spanning 0–10 points that measures capacity to complete an important activity that the client is unable to do or having difficulty performing as a result of their reason for presentation. Zero represents an inability to perform the activity, whereas 10 signifies capacity to perform the activity at the same level as before the reason for presentation occurred. The patient-specific functional scale has good test–retest reliability (ICC = 0.82) [14].

#### Pain Intensity

The numeric pain rating scale is three single-item scales spanning 0–10 points that measures current, worst in the last week and best in the last week pain intensity [15]. Zero represents no pain, and 10 signifies the worst pain imaginable. The numeric pain rating scale has moderate-excellent test–retest reliability (ICC = 0.67–0.96) [16].

#### Goal Achievement

The goal attainment scaling is a method for scoring the extent to which a client identified goal is achieved [17]. The method allows for differing goals to be standardised for aggregated analyses via calculation of t-scores and is commonly implemented in the rehabilitation field [18].

#### Adverse Events

Serious adverse events were defined as any untoward medical occurrent that results in death, is life threatening or requires hospitalisation [19]. Non-serious adverse events were defined as any other untoward medical occurrent [19]. Adverse events were classified as treatment-related if they were definitely, possibly or probably related to the provision of exercise physiology services [19].

### Valuation of Costs

All costs were calculated in Australian dollars (AUD) using 2020 as a base year. Direct resource costs included the staff time required for either in-person or telehealth delivery of exercise physiology services and were valued using local wage rates (grade 2, year 4 exercise physiologist; equivalent to a private allied health professional with 4 years of experience) multiplied by 1.3 to cover employment oncosts (AUD63.04 per hour). Physical resources, such as clinic rooms, and standard office resources, such as computers and printers, were in place prior to the service commencement and therefore these costs were excluded from all analysis. The value of each in-person or telehealth delivery services assumed purchase (minus patient out of pocket expense) through the Medicare Benefits Schedule (AUD55.08 per session), National Disability Insurance Scheme (AUD166.99 per session) and Department of Veteran Affairs (AUD66.90 per session). Participants who missed EQ-5D-5L follow-up appointments were not included in the economic evaluation. Due to participant data entry errors, three participants did not have direct healthcare costs provided and a conservative value of AUD0.00 was imputed for these participants.

### Statistical Analyses

All analyses were conducted using Stata (17, StataCorp, College Station, TX). Linear mixed models with random effects (clients) determined within- and between-group (delivery mode) differences in client-reported outcome measures over time. Sensitivity analyses considered the aforementioned model with additional fixed effects for treatment duration or adherence. The financial costs of exercise physiology services by delivery mode were conducted from the health service perspective, and client out-of-pocket costs were not included. Healthcare costs over the follow-up period were summed within individual participants. A total cost variable was calculated for each participant who provided healthcare costs by summing all cost domains across the follow-up time horizon by delivery modes. All costs were compared between groups using both mean (SD) and median (IQR) to account for varying distribution of data. An alpha of 0.05 was adopted for all analyses.

## Results

### Participants

#### Clinicians

Client datasets were provided by five individual clinicians and four clinics (see Sect. 2.3). The median (range) number of client datasets provided was 2 (1–6) datasets from individual clinicians and 22 (7–75) datasets from clinics. Among participating individual clinicians, client datasets (*n* = 12) indicated 42% (*n* = 5) received telehealth delivery only, 42% (*n* = 5) received a combination of in-person and telehealth delivery and 17% (*n* = 2) received in-person delivery only. Among participating clinics, client datasets (*n* = 126) indicated 63% (*n* = 80) received a combination of in-person and telehealth delivery, 32% (*n* = 40) received in-person delivery only and 5% (*n* = 6) received telehealth delivery only.

#### Clients

Collectively, 138 client datasets (female: *n* = 69, 50%) were provided by participating clinicians. Client geographical location spanned 89 unique postcodes and six states/territories (Table 1). Victoria was the most common location of residence (43%), followed by Western Australia (33%) and New South Wales (14%). The most common age range was 55–64 years (28%), followed by 45–54 years (23%) and 35–44 years (19%). Of the 17% of overall client datasets that included body mass index, 83% of clients were overweight or obese. Most clients were referred privately (49%) or via WorkCover (41%). Musculoskeletal disease (77%) was the most common reason for seeking exercise physiology services, followed by multimorbidity (6%) and cardiovascular disease (6%).

**Table 1:** Client baseline demographics

Variable	Clients (*n* = 138)
**Sex, *n* (%)**	
Female	69 (50.0)
Male	69 (50.0)
**State/territory of residence, *n* (%)**	
Victoria	60 (43.5)
Western Australia	46 (33.3)
New South Wales	19 (13.8)
Queensland	8 (5.8)
Northern Territory	4 (2.9)
Australian Capital Territory	1 (0.7)
**Age, *n* (%)**	
18–24 years	4 (2.9)
25–34 years	11 (8.0)
35–44 years	26 (18.8)
45–54 years	32 (23.2)
55–64 years	38 (27.5)
65–74 years	20 (14.5)
75–84 years	5 (3.6)
Not reported	2 (1.5)
**Body mass index, *n* (%)**	
< 25 kg/m^2^	4 (2.9)
25–29.9 kg/m^2^	6 (4.4)
30–34.9 kg/m^2^	9 (6.5)
35–39.9 kg/m^2^	2 (1.5)
40–44.9 kg/m^2^	1 (0.7)
> 45 kg/m^2^	1 (0.7)
Not reported	115 (83.3)
**Source of referral, *n* (%)**	
Private	67 (48.6)
WorkCover	56 (40.6)
Medicare	10 (7.3)
Department of Veterans' Affairs	3 (2.2)
National Disability Insurance Scheme	2 (1.5)
**Condition, *n* (%)**	
Musculoskeletal	106 (76.8)
Multiple conditions	8 (5.8)
Cardiovascular	8 (5.8)
Neurological	6 (4.4)
Type 2 diabetes	3 (2.2)
Cancer/post-cancer	2 (1.5)
Obesity/metabolic (not type 2 diabetes)	2 (1.5)
Other	2 (1.5)
Mental health	1 (0.7)

Data are count (percentage within-group)

### Exercise Physiology Services

Most (59%, *n* = 82) clients received treatment implemented via in-person delivery only, whereas 8% (*n* = 11) utilised telehealth delivery only and 33% (*n* = 45) were provided via implementation of a combination of telehealth and in-person delivery. Mean (SD) treatment duration, regardless of delivery mode, was 11 (7) weeks (range: 2–52 weeks) and included 12 (6) sessions (range: 2–30 sessions) lasting 48 (9) minutes (range: 30–60 min). Mean (SD) treatment duration for telehealth delivery only was 16 (9) weeks (range: 7–30 weeks) and included 11 (9) sessions (range: 2–30 sessions) lasting 45 (15) minutes (range: 30–60 min). Mean (SD) treatment duration for in-person delivery only was 9 (3) weeks (range: 2–16 weeks) and included 10 (5) sessions (range: 2–24 sessions) lasting 45 (5) minutes (range: 30–60 min). Mean (SD) duration for treatment implemented with combined in-person and telehealth delivery was 13 (10) weeks (range: 6–52 weeks) and included 15 (7) sessions (range: 2–30 sessions) lasting 54 (9) minutes (range: 30–60 min). The majority (73%) of clients completed > 80% of allocated treatment. Client treatment adherence did not differ by implementation delivery mode (*P* = 0.489).

### Effectiveness

#### Any Delivery Mode

**Fig. 1:**
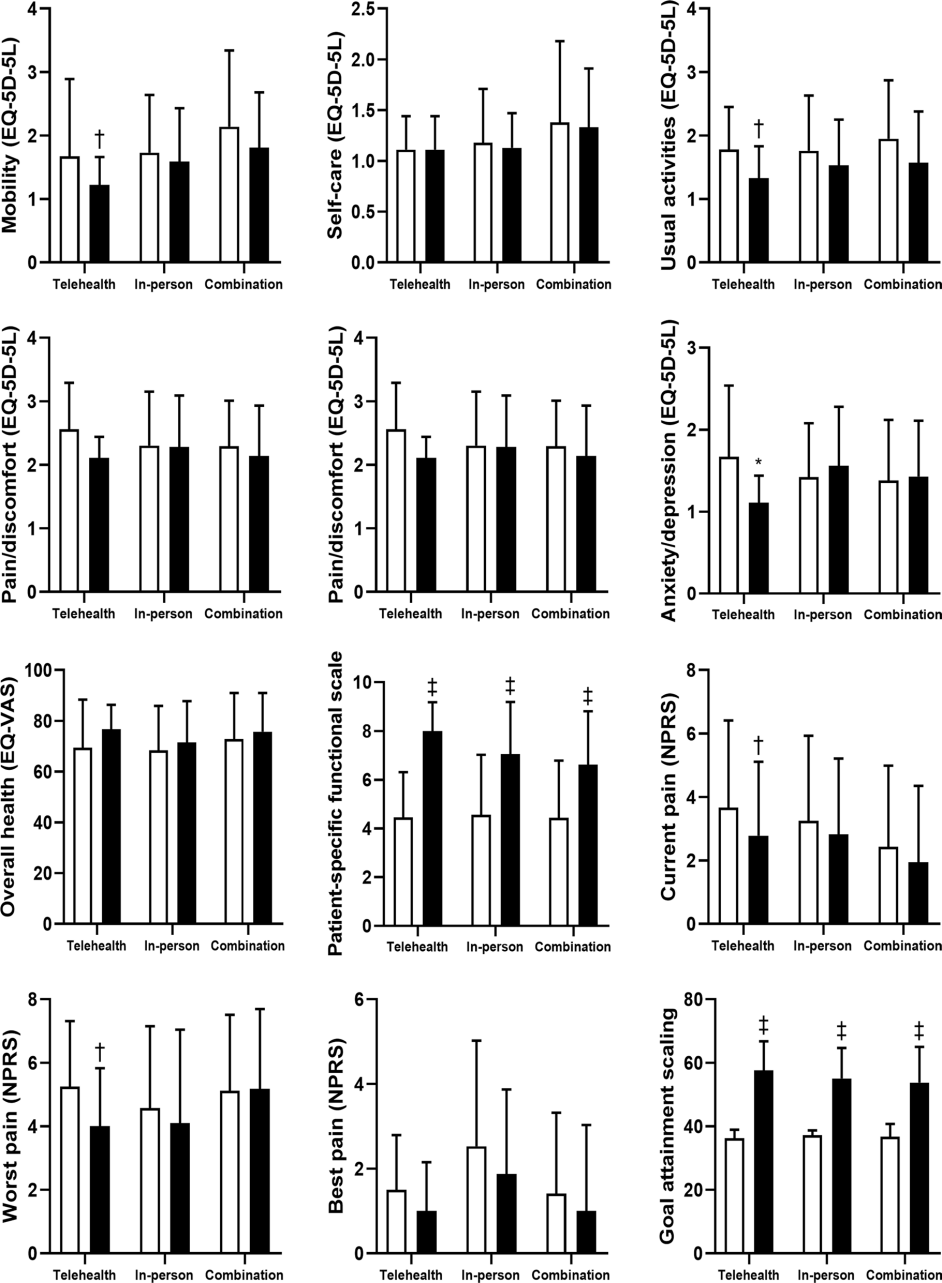
Effects of an exercise physiology treatment phase on patient outcomes by delivery mode. Telehealth: telehealth delivery only (*n* = 11), In-person: in-person delivery only (*n* = 82), Combination: combination of telehealth and in-person delivery (*n* = 45). *P < 0.05; ^†^*P* < 0.01; ^‡^*P* < 0.001 compared to baseline. White bar: Baseline, Black bar: Follow-up

Exercise physiology via any delivery mode improved mobility (EQ-5D-5L) by 14% (*β* = − 0.23, *P* = 0.003), capacity to complete usual activities (EQ-5D-5L) by 18% (*β* = − 0.29, *P* < 0.001), capacity to complete important activities that the client was unable to do or having difficulty performing as a result of their reason for presentation (patient-specific functional scale) by 54% (*β* = 2.46, *P* < 0.001), current pain intensity (numeric pain rating scale) by 16% (*β* = − 0.55, *P* = 0.038) and goal attainment scaling t-scores by 50% (*β* = 18.37, *P* < 0.001; Table 2; Fig. 1).

**Table 2:** Effects of an exercise physiology treatment phase on patient outcomes

	Any delivery (*n* = 138)		Telehealth delivery only (*n* = 11)		In-person delivery only (*n* = 82)		Combination of telehealth and in-person delivery (*n* = 45)	
	Baseline	Follow-up	Baseline	Follow-up	Baseline	Follow-up	Baseline	Follow-up
Mobility (EQ-5D-5L)	1.86 (1.01)	1.61 (0.82)^†^	1.67 (0.87)	1.22 (0.44)^†^	1.73 (0.91)	1.59 (0.84)	2.14 (1.20)	1.81 (0.87)*
Self-care (EQ-5D-5L)	1.24 (0.61)	1.19 (0.44)	1.11 (0.33)	1.11 (0.33)	1.18 (0.53)	1.13 (0.34)	1.38 (0.80)	1.33 (0.58)
Usual activities (EQ-5D-5L)	1.83 (0.85)	1.52 (0.72)^‡^	1.78 (0.67)	1.33 (0.50)^†^	1.76 (0.87)	1.53 (0.72)	1.95 (0.92)	1.57 (0.81)^†^
Pain/discomfort (EQ-5D-5L)	2.33 (0.78)	2.21 (0.75)	2.56 (0.73)	2.11 (0.33)	2.30 (0.85)	2.28 (0.81)	2.29 (0.72)	2.14 (0.79)
Anxiety/depression (EQ-5D-5L)	1.44 (0.71)	1.45 (0.67)	1.67 (0.87)	1.11 (0.33)*	1.42 (0.66)	1.56 (0.72)	1.38 (0.74)	1.43 (0.68)
Utility score (EQ-5D-5L)	0.72 (0.15)	0.75 (0.12)	0.70 (0.14)	0.79 (0.06)	0.73 (0.16)	0.74 (0.14)	0.72 (0.15)	0.75 (0.13)
Overall health (EQ-VAS)	70.03 (17.72)	73.68 (15.09)	69.44 (18.94)	76.67 (9.67)	68.36 (17.52)	71.47 (16.28)	72.90 (18.04)	75.76 (15.18)
Patient-specific functional scale	4.51 (2.37)	6.99 (2.11)^‡^	4.45 (1.86)	8.00 (1.18)^‡^	4.56 (2.46)	7.06 (2.13)^‡^	4.44 (2.35)	6.62 (2.19)^‡^
Current pain (NPRS)	3.03 (2.64)	2.52 (2.38)*	3.67 (2.74)	2.78 (2.33)^‡^	3.25 (2.68)	2.82 (2.39)	2.43 (2.56)	1.95 (2.40)
Worst pain (NPRS)	4.80 (2.46)	4.44 (2.74)	5.25 (2.06)	4.00 (1.83)^‡^	4.57 (2.58)	4.10 (2.94)	5.12 (2.39)	5.18 (2.51)
Best pain (NPRS)	2.08 (2.27)	1.52 (1.98)	1.50 (1.29)	1.00 (1.15)	2.53 (2.49)	1.87 (2.00)	1.41 (1.91)	1.00 (2.03)
Goal attainment scaling	36.90 (2.77)	55.00 (10.03)^‡^	36.22 (2.73)	57.58 (9.18)^‡^	37.21 (1.52)	55.07 (9.61)^‡^	36.71 (4.06)	53.72 (11.27)^‡^

Data are mean (SD). NPRS: Numeric pain rating scale. *P < 0.05; ^†^*P* < 0.01; ^‡^*P* < 0.001 compared to baseline

#### In-Person Delivery Only

Exercise physiology via in-person delivery improved capacity to complete important activities that the client was unable to do or having difficulty performing as a result of their reason for presentation (patient-specific functional scale) by 55% (*β* = 2.49, *P* < 0.001) and goal attainment scaling t-scores by 48% (*β* = 17.86, *P* < 0.001; Table 2; Fig. 1.

#### Telehealth Delivery Only

Exercise physiology via telehealth delivery improved mobility (EQ-5D-5L) by 27% (*β* = − 0.44, *P* = 0.007), capacity to complete usual activities (EQ-5D-5L) by 25% (*β* = − 0.44, *P* = 0.007), anxiety/depression (EQ-5D-5L) by 33% (*β* = − 0.56, *P* = 0.015), capacity to complete important activities that the client was unable to do or having difficulty performing as a result of their reason for presentation (patient-specific functional scale) by 80% (*β* = 3.55, *P* < 0.001), current pain intensity (numeric pain rating scale) by 24% (*β* = − 0.89, *P* = 0.002), worst pain intensity (numeric pain rating scale) by 24% (*β* = − 1.25, *P* = 0.003) and goal attainment scaling t-scores by 59% (*β* = 21.36, *P* < 0.001; Table 2; Fig. 1).

#### Combination of In-Person and Telehealth Delivery

Exercise physiology via a combination of in-person and telehealth delivery improved mobility (EQ-5D-5L) by 16% (*β* = − 0.35, *P* = 0.043), capacity to complete usual activities (EQ-5D-5L) by 21% (*β* = − 0.40, *P* = 0.002), capacity to complete important activities that the client was unable to do or having difficulty performing as a result of their reason for presentation (patient-specific functional scale) by 46% (*β* = 2.15, *P* < 0.001) and goal attainment scaling t-scores by 48% (*β* = 17.81, *P* < 0.001; Table 2; Fig. 1).

#### Sensitivity Analyses

Results did not differ for any outcome or delivery mode (any, in-person, telehealth or combination) when models accounted for variance in treatment duration or adherence.

#### Differences Between Delivery Modes

No differences in client-reported outcomes were observed between delivery modes for mobility (EQ-5D-5L; *P* = 0.087), self-care (EQ-5D-5L; *P* = 0.119), usual activities (EQ-5D-5L; *P* = 0.476), pain/discomfort (EQ-5D-5L; *P* = 0.618), anxiety/depression (EQ-5D-5L; *P* = 0.899), utility score (EQ-5D-5L; *P* = 0.900), overall health (EQ-VAS; *P* = 0.582), patient-specific functional scale (*P* = 0.259), current pain (NPRS; *P* = 0.116), worst pain (NPRS; *P* = 0.347), best pain (NPRS; *P* = 0.299) or goal attainment scaling (*P* = 0.387; Fig. 1; Table 2).

### Adverse Events

No serious adverse events were reported. One non-serious treatment-related adverse event (low-grade soreness post-exercise session) was reported for in-person delivery.

### Valuation of Costs

Comparison of crude costs (AUD in 2020) accrued over the intervention follow-up between exercise physiology delivery modes is shown in Table 3. The total crude cost of providing the combined in-person and telehealth delivery and any delivery follow-up approach was approximately AUD700.00–800.00 greater per client than either the telehealth only or in-person only delivery approach. This was largely driven by National Disability Insurance Scheme costs with mean (SD) difference per client provided the combined delivery of AUD429.40 (AUD1,365.41) and any delivery of AUD145.44 (AUD808.23). The difference between delivery modes in this line item was highly skewed (all delivery modes: median [IQR]: AUD0.00 [AUD0.00]) indicating a relatively small number of clients contributed large cost values to the overall evaluation of this outcome. Sensitivity analyses that removed the three participants who did not have measurements of direct healthcare costs due to administrative error did not alter total costs when considering any delivery modality; however, telehealth delivery specifically was approximately AUD300.00 greater per client (Additional file 1: Table S1).

**Table 3:** Crude comparison of costs (AUD at 2020) accrued over the intervention follow-up between delivery modes

Cost domain	Any delivery (*n* = 62)	In-person delivery only (*n* = 32)	Telehealth delivery only (*n* = 9)	Combination of telehealth and in-person delivery (*n* = 21)
	Mean (SD) Median (IQR)	Mean (SD) Median (IQR)	Mean (SD) Median (IQR)	Mean (SD) Median (IQR)
Department Veteran Affairs benefits paid	6.47 (50.98) 0.00 (0.00, 0.00)	12.54 (70.96) 0.00 (0.00, 0.00)	0.00 (0.00) 0.00 (0.00, 0.00)	0.00 (0.00) 0.00 (0.00, 0.00)
Medical Benefits Scheme benefits paid	79.95 (225.82) 0.00 (0.00, 0.00)	82.62 (230.73) 0.00 (0.00, 0.00)	0.00 (0.00) 0.00 (0.00, 0.00)	110.16 (263.00) 0.00 (0.00, 0.00)
National Disability Insurance Scheme benefits paid	145.44 (808.23) 0.00 (0.00, 0.00)	0.00 (0.00) 0.00 (0.00, 0.00)	0.00 (0.00) 0.00 (0.00, 0.00)	429.40 (1365.41) 0.00 (0.00, 0.00)
Direct resource costs	824.08 (499.89) 787.50 (425.25, 1149.75)	708.75 (330.67) 661.50 (441.00, 1008.00)	714.00 (692.28) 504.00 (0.00, 1228.50)	1047.00 (567.52) 882.00 (7630.00, 1480.50)
Total costs^a^	1055.95 (1089.64) 882.00 (441.00, 1260.00)	803.91 (417.04) 756.00 (441.00, 1008.00)	714.00 (692.28) 504.00 (0.00, 1228.50)	1586.56 (1646.24) 1260.00 (630.00, 1771.56)

^a^Total cost includes the sum of all costs: Department of Veteran Affairs benefits paid, Medical Benefits Scheme benefits paid, National Disability Insurance Scheme benefits paid and direct resource cost

## Discussion

The results of the current study showed exercise physiology services provided during the COVID-19 pandemic: (1) improved mobility and capacity to complete usual activities (EQ-5D-5L), (2) increased capacity to complete important activities that clients were unable to do or had difficulty performing at baseline (patient-specific functional scale), (3) reduced current pain intensity (numeric pain rating scale), and (4) resulted in clients achieving their treatment-based goals. Importantly, no differences were observed between delivery modalities (i.e. telehealth only, in-person only, combination of telehealth and in-person) for client-reported outcomes of treatment effectiveness.

The current study showed that exercise physiology services provided during the COVID-19 pandemic, regardless of delivery modality, led to improvements in a range of client-reported outcomes commonly implemented in clinical practice. These observations align with pre-pandemic evidence that exercise physiology services can improve client-reported outcomes across a broad range of clinical population groups [6]. Our findings support the notion that despite a shift in delivery mode borne from the COVID-19 pandemic, exercise physiology services remained effective at improving client health-based outcomes. Determining whether these effects are clinically meaningful is therefore pertinent.

Our study detected clinically meaningful changes in capacity to complete important activities that clients were unable to do or had difficulty performing at baseline [20] and goal attainment [18]. In contrast, reduction in current pain intensity among clients in our study failed to reach clinically meaningful thresholds (− 1.5 points) [20], which likely stemmed from the low mean pain intensity and marked negative skewing of values (26% of clients reported no pain at baseline). Moreover, mean change in utility score (EQ-5D-5L) in our study did not meet the established clinically meaningful value (0.037) [21]. Notably, the overall health (EQ-VAS) of clients in the current study was within 0.5 SD of Australian population norms [22], which may have influenced our capacity to detect clinically meaningful improvements. Collectively, our findings support that exercise physiology services provided during the COVID-19 pandemic were capable of achieving clinically meaningful improvements in some, yet not all, client-reported outcomes. Given we did not identify differences in effectiveness by delivery mode, our observations support the ongoing investigation of less traditional delivery of exercise physiology (e.g. telehealth delivery) given the potential for these modes to accommodate the one in five clients that prefer access to allied health services via telehealth delivery [10].

A key strength of the current study was the ecological validity borne from pragmatic observations of exercise physiology services. However, our study was not without limitation. First, the vast majority of clients included had musculoskeletal conditions and thus observations may be less generalisable to other clinical population groups that commonly utilise exercise physiology services. Second, data only represented six of the eight Australian states and territories and approximately three quarters of clients resided in Victoria or Western Australia. Third, government COVID-19 restrictions differed between states and territories. For example, stay-at-home orders were in effect within Victoria for 113 days during our data collection period, whereas Western Australia had no stay-at-home orders during this period. Fourth, between-group differences based on delivery modality should be interpreted with caution given unbalanced sample sizes. Subsequently, these uneven samples in conjunction with our overall sample size precluded our capacity to conduct statistically powered equivalence testing (e.g. via two one-sided t-test). Factors contributing to this were the overall sample size and the highly skewed nature of some cost variables. To date, no economic evaluation has been published examining the comparative cost-effectiveness of these three approaches in a private practise setting. Future economic evaluation using comparative, trial-based, incremental cost-utility analysis conducted from the societal perspective is required. Therefore, we cannot definitively conclude that these modalities are equivalent. Fifth, as we only collected data during the COVID-19 pandemic, direct comparisons to pre-pandemic treatment efficacy was not possible. Finally, a limitation of our cost evaluation was that we did not capture direct and indirect healthcare and productivity costs. Whilst we would expect a potential cost productivity saving from the client perspective in terms of travel and time off work for the telehealth delivery follow-up approach, research conducting a comparative incremental cost-utility of the three approaches examined in the current study is warranted to further inform clinical decision-making in this area.

## Conclusion

Exercise physiology services provided during the COVID-19 pandemic increased functional capacity, reduced pain intensity and resulted in clients achieving their treatment-based goals. Changes in client-reported outcome measures did not differ by delivery mode. Studies explicitly designed to evaluate comparative cost-effectiveness of exercise physiology service delivery modalities and long-term maintenance of implementing these approaches are warranted.

## Supplementary Information

**Additional file 1. Supplementary Table S1.** Sensitivity analysis of the crude comparison of costs (AUD at 2020) accrued over the intervention follow-up between delivery modes.

## Data Availability

Data available on request from the authors.

## References

[1] Jones NR, Qureshi ZU, Temple RJ, Larwood JPJ, Greenhalgh T, Bourouiba L (2020). Two metres or one: what is the evidence for physical distancing in COVID-19?. BMJ.

[2] Malliaras P, Merolli M, Williams CM, Caneiro JP, Haines T, Barton C (2021). ‘It’s not hands-on therapy, so it’s very limited’: telehealth use and views among allied health clinicians during the coronavirus pandemic. Musculoskelet Sci Pract.

[3] Cortez C, Mansour O, Qato DM, Stafford RS, Alexander GC (2021). Changes in short-term, long-term, and preventive care delivery in US office-based and telemedicine visits during the COVID-19 pandemic. JAMA Health Forum.

[4] Owen PJ, Keating SE, Askew CD, Clanchy KM, Jansons P, Maddison R (2022). Impact of the covid-19 pandemic on exercise physiology services in Australia: a retrospective audit. Sports Med Open.

[5] Exercise and Sports Science Australia (2018). Accredited exercise physiologist scope of practice.

[6] Pedersen BK, Saltin B (2015). Exercise as medicine—evidence for prescribing exercise as therapy in 26 different chronic diseases. Scand J Med Sci Sports.

[7] Owen PJ, Miller CT, Mundell NL, Verswijveren SJJM, Tagliaferri SD, Brisby H (2020). Which specific modes of exercise training are most effective for treating low back pain? Network meta-analysis. Br J Sports Med.

[8] Saueressig T, Pedder H, Bowe SJ, Owen PJ, Belavy DL (2021). Six meta-analyses on treatments for femoroacetabular impingement syndrome in a year and readers are none the wiser: methods advice for researchers planning meta-analysis of data from fewer than 5 trials. J Orthop Sports Phys Ther.

[9] Costa RRG, Dorneles JR, Veloso JH, Gonçalves CW, Neto FR (2021). Synchronous and asynchronous tele-exercise during the coronavirus disease 2019 pandemic: comparisons of implementation and training load in individuals with spinal cord injury. J Telemed Telecare.

[10] Filbay S, Hinman R, Lawford B, Fry R, Bennell K (2021). Telehealth by allied health practitioners during the COVID-19 pandemic: an Australian wide survey of clinicians and clients.

[11] Herdman M, Gudex C, Lloyd A, Janssen M, Kind P, Parkin D (2011). Development and preliminary testing of the new five-level version of EQ-5D (EQ-5D-5L). Qual Life Res.

[12] van Hout B, Janssen MF, Feng Y-S, Kohlmann T, Busschbach J, Golicki D (2012). Interim scoring for the EQ-5D-5L: mapping the EQ-5D-5L to EQ-5D-3L value sets. Value Health.

[13] Long D, Polinder S, Bonsel GJ, Haagsma JA (2021). Test–retest reliability of the EQ-5D-5L and the reworded QOLIBRI-OS in the general population of Italy, the Netherlands, and the United Kingdom. Qual Life Res.

[14] Mathis RA, Taylor JD, Odom BH, Lairamore C (2019). Reliability and validity of the patient-specific functional scale in community-dwelling older adults. J Geriatr Phys Ther.

[15] Downie WW, Leatham PA, Rhind VM, Wright V, Branco JA, Anderson JA (1978). Studies with pain rating scales. Ann Rheum Dis.

[16] Kahl C, Cleland JA (2005). Visual analogue scale, numeric pain rating scale and the McGill pain questionnaire: an overview of psychometric properties. Phys Ther Rev.

[17] Kiresuk TJ, Sherman RE (1968). Goal attainment scaling: a general method for evaluating comprehensive community mental health programs. Community Ment Health J.

[18] Turner-Stokes L (2009). Goal attainment scaling (GAS) in rehabilitation: a practical guide. Clin Rehabil.

[19] Ioannidis JPA, Evans SJW, Gøtzsche PC, O’Neill RT, Altman DG, Schulz K (2004). Better reporting of harms in randomized trials: an extension of the CONSORT statement. Ann Intern Med.

[20] Abbott JH, Schmitt J (2014). Minimum important differences for the patient-specific functional scale, 4 region-specific outcome measures, and the numeric pain rating scale. J Orthop Sports Phys Ther.

[21] McClure NS, Sayah FA, Xie F, Luo N, Johnson JA (2017). Instrument-defined estimates of the minimally important difference for EQ-5D-5L index scores. Value Health.

[22] McCaffrey N, Kaambwa B, Currow DC, Ratcliffe J (2016). Health-related quality of life measured using the EQ-5D–5L: South Australian population norms. Health Qual Life Outcomes.

