# Supplementary material for: The Effectiveness of Exercise Physiology Services During the COVID-19 Pandemic: A Pragmatic Cohort Study
Source: Sports Med Open. 2023 Jan 8;9:2. doi: 10.1186/s40798-022-00539-3 (PMC9826725; doi:10.1186/s40798-022-00539-3)
Supplement: Supplementary file 1 — Additional file 1. Supplementary Table S1. Sensitivity analysis of the crude comparison of costs (AUD at 2020) accrued over the intervention follow-up between delivery modes. [file 40798_2022_539_MOESM1_ESM.docx]

**Supplementary Table S1**. Sensitivity analysis of the crude comparison of costs (AUD at 2020) accrued over the intervention follow-up between delivery modes.

| Cost domain | Any delivery (n=59) | In-person delivery only (n=32) | Telehealth delivery only (n=6) | Combination of telehealth and in-person delivery (n=21) |
| --- | --- | --- | --- | --- |
|  | Mean (SD)  Median (IQR) | Mean (SD)  Median (IQR) | Mean (SD)  Median (IQR) | Mean (SD)  Median (IQR) |
| Department Veteran Affairs benefits paid | 6.80 (52.25)  0.00 (0.00, 0.00) | 12.54 (70.96)  0.00 (0.00, 0.00) | 0.00 (0.00)  0.00 (0.00, 0.00) | 0.00 (0.00)  0.00 (0.00, 0.00) |
| Medical Benefits Scheme benefits paid | 84.02 (230.83)  0.00 (0.00, 0.00) | 82.62 (230.73)  0.00 (0.00, 0.00) | 0.00 (0.00)  0.00 (0.00, 0.00) | 110.16 (263.00)  0.00 (0.00, 0.00) |
| National Disability Insurance Scheme benefits paid | 152.83 (828.17)  0.00 (0.00, 0.00) | 0.00 (0.00)  0.00 (0.00, 0.00) | 0.00 (0.00)  0.00 (0.00, 0.00) | 429.40 (1365.41)  0.00 (0.00, 0.00) |
| Direct resource costs | 865.98 (475.29) 882.00 (441.00, 1197.00) | 708.75 (330.67)  661.50 (441.00, 1008.00) | 1071.00 (554.97)  1197.00 (472.5, 1417.5) | 1047.00 (567.52)  882.00 (7630.00, 1480.50) |
| Total costs ^a^ | 1109.64 (1090.00) 882.00 (441.00, 1260.00) | 803.91 (417.04)  756.00 (441.00, 1008.00) | 1071.00 (554.97)  1197.00.00 (472.5, 1417.5) | 1586.56 (1646.24)  1260.00 (630.00, 1771.56) |

^a^ Total cost includes the sum of all costs: Department of Veteran Affairs benefits paid, Medical Benefits Scheme benefits paid, National Disability Insurance Scheme benefits paid and direct resource cost.
